# Evolution of promoter-proximal pausing enabled a new layer of transcription control

**DOI:** 10.21203/rs.3.rs-2679520/v1

**Published:** 2023-03-24

**Authors:** Alexandra G. Chivu, Abderhman Abuhashem, Gilad Barshad, Edward J. Rice, Michelle M. Leger, Albert C. Vill, Wilfred Wong, Rebecca Brady, Jeramiah J. Smith, Athula H. Wikramanayake, César Arenas-Mena, Ilana L. Brito, Iñaki Ruiz-Trillo, Anna-Katerina Hadjantonakis, John T. Lis, James J. Lewis, Charles G. Danko

**Affiliations:** 1 Baker Institute for Animal Health, College of Veterinary Medicine, Cornell University, Ithaca, NY 14853, USA.; 2 Department of Molecular Biology & Genetics, Cornell University, Ithaca, NY 14853, USA.; 3 Department of Biomedical Sciences, College of Veterinary Medicine, Cornell University, Ithaca, NY 14853, USA.; 4 Developmental Biology Program, Sloan Kettering Institute, Memorial Sloan Kettering Cancer Center, NY 10065, USA.; 5 Weill Cornell/Rockefeller/Sloan Kettering Tri-Institutional MD-PhD Program, NY 10065, USA.; 6 Biochemistry Cell and Molecular Biology Program, Weill Cornell Graduate School of Medical Sciences, Cornell University, NY 10065, USA.; 7 Department of Biology at the College of Staten Island and PhD Programs in Biology and Biochemistry at The Graduate Center, The City University of New York (CUNY), Staten Island, NY 10314, USA.; 8 Department of Genetics and Biochemistry, Clemson University, 105 Collings St, Clemson, SC 29634.; 9 Department of Biology, University of Kentucky, Lexington, KY, 40506, USA.; 10 Institute of Evolutionary Biology (CSIC-Universitat Pompeu Fabra), Barcelona, 08003, Spain.; 11 ICREA, Pg. Lluis Companys 23, 08010 Barcelona, Spain., Barcelona, 08003, Spain.; 12 Meinig School of Biomedical Engineering, Cornell University, Ithaca, NY 14850, USA.; 13 Computational and Systems Biology Program, Memorial Sloan Kettering Cancer Center, New York, NY 10065, USA.; 14 Tri-Institutional training Program in Computational Biology and Medicine, New York, NY 10065, USA.; 15 Department of Biology, University of Miami, Coral Gables, FL 33146.; 16 Department of Biology, Ithaca College, Ithaca NY 14850, USA

## Abstract

Promoter-proximal pausing of RNA polymerase II (Pol II) is a key regulatory step during transcription. Despite the central role of pausing in gene regulation, we do not understand the evolutionary processes that led to the emergence of Pol II pausing or its transition to a rate-limiting step actively controlled by transcription factors. Here we analyzed transcription in species across the tree of life. We found that unicellular eukaryotes display a slow acceleration of Pol II near transcription start sites. This proto-paused-like state transitioned to a longer, focused pause in derived metazoans which coincided with the evolution of new subunits in the NELF and 7SK complexes. Depletion of NELF reverts the mammalian focal pause to a proto-pause-like state and compromises transcriptional activation for a set of heat shock genes. Collectively, this work details the evolutionary history of Pol II pausing and sheds light on how new transcriptional regulatory mechanisms evolve.

## Introduction

**Figure 1:**
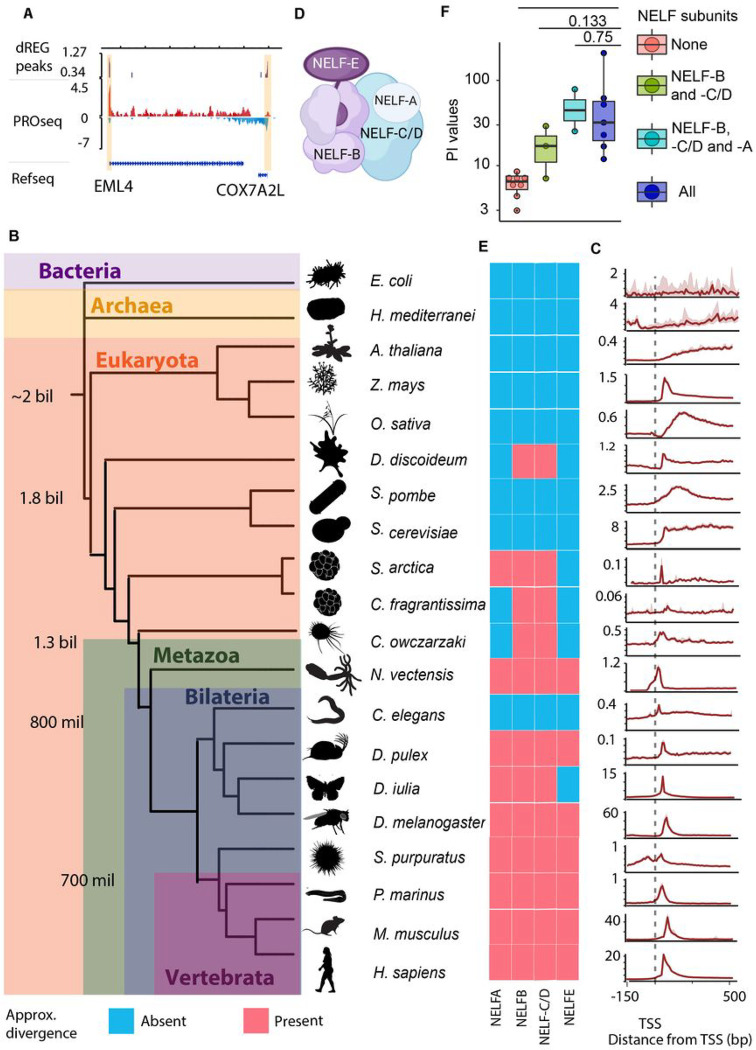
The evolution of NELF subunits is associated with pausing. (A) Depiction of a PRO-seq track where red represents sense and blue antisense transcription. dREG peaks are marked in purple and pause regions are highlighted in yellow. (B) Schematic depicting the relationships between the 20 species included in this study. Divergence times were taken from ^41^ and ^42^. (C) Meta profiles of PRO-seq data were collected in each species. The dotted line marks the position of TSSs. The 25–75% confidence intervals are depicted in transparent red. (D) Cartoon depicting internal interactions in the NELF complex based on the crystal structure in ^21^. (E) Colored blocks denote the presence (red) or absence (blue) of the human orthologues of NELF subunits in each species as inferred from reciprocal blast searches. (F) Box and whiskers plot of pausing indexes in each species. Boxes are clustered by the number of NELF subunits in each species. A Mann-Whitney test was used to compute p-values.

The evolution of complex transcriptional regulatory programs is one of the defining characteristics of metazoans which enables the organismal complexity required for animal development. “Pausing” is one of the regulatory stages during transcription by RNA Polymerase II (Pol II). Pol II transiently “pauses” 20–60 bases downstream of the transcription start site (TSS) at all genes in *Drosophila* and mammals, disrupting the continuous flow of transcription (Fig. 1A)^1–4^. The rate at which polymerases are “released” from a paused state into productive elongation is actively regulated by transcription factors^5^, and is therefore essential for proper development in most animal species^6–8^. However, unicellular model organisms, including yeast^9,10^, do not have a promoter-proximal pause. To date, no study has characterized the distribution of Pol II outside of a few key model organisms, leaving when and how the pause evolved as an open question.

## Results

### NELF subunit evolution increased the residence time of Pol II in a pause state

We used Precision Run-On and Sequencing (PRO-seq)^11^ to study transcription in 20 extant organisms that represent two billion years of evolution (Fig. 1B), including multiple species near the base of the Metazoa phylogeny and the transition between plants and animals. Our atlas of organisms adds new data representing two prokaryotic organisms (*Escherichia coli* and *Haloferax mediterranei*, representing the bacteria and archaea domains, respectively), and single-celled eukaryotes including the social amoeba (*Dictyostelium discoideum*), two ichthyosporeans (*Creolimax fragrantissima,* and *Sphaeroforma arctica*), and a filasterean (*Capsaspora owczarzaki*). We also included a number of metazoan organisms representing major taxa, including the cnidarian (*Nematostella vectensis*), the sea urchin (*Strongylocentrotus purpuratus*), the water flea (*Daphnia pulex*), the butterfly (*Dryas iulia*), and the cyclostome (*Petromyzon marinus*). Finally, we have augmented our PRO-seq atlas by integrating published data from a fly (*Drosophyla melanogaster*^11^), a nematode (*Caenorhabditis elegans*^12^), yeast (Saccharomyces *cerevisia*e and *Schizosaccharomyces pombe*^9,10^), model plants (*Arabidopsis thaliana*, *Oryza sativa*, and *Zea mays*^13–15^), and mammals (*Homo sapiens* and *Mus musculus*^7,16^). These species occupy key positions along the phylogenetic tree, allowing us to investigate most major transitions in the animal lineage.

As expected, most metazoan organisms exhibited a pileup of RNA polymerase 30–100 base pairs downstream of the TSS indicative of Pol II promoter-proximal pausing (Fig. 1C). Conversely, prokaryotic organisms lacked a prominent Pol II peak in our data. Plants and unicellular eukaryotes exhibited more diverse pause variation: the plant *Z. mays* showed a focused peak, while the plant *O. sativa* and the yeast *S. pombe* displayed a more dispersed peak downstream of the TSS. The plant *A. thaliana* and yeast *S. cerevisiae* showed no evidence of pausing. To reveal more subtle differences in the dynamics and gene-by-gene variation at the pause site than observed in meta profiles, we computed pausing indexes which quantify the duration Pol II spends in a promoter-proximal paused state^3,17^. Pausing indexes revealed that the residence time of Pol II at the pause site is, on average, 1–2 orders of magnitude higher in metazoans than unicellular eukaryotes or plants (**fig. S1**). Consistent with the meta plots, we also noted wide variation in pausing indices in unicellular eukaryotes and plants. Taken together, our results suggest that an ancestral slowdown in Pol II transcription near the TSS may have arisen in unicellular eukaryotes and became longer in duration and more focused during the early evolution of metazoans.

Pausing is mediated by interactions between Pol II, DRB sensitivity inducing factor (DSIF), and the negative elongation factor (NELF) complex^18,19^. Of these proteins, the NELF complex can establish pausing both *in vitro* and *in vivo*^20^. The NELF complex consists of multiple subunits, including NELF-A, -B, -C (or its isoform -D), and -E. CryoEM studies revealed that NELF-B and -E form a sub-complex, while NELF-C/-D and -A form a separate subcomplex. NELF-B and -C/-D interact with one another forming a core structure that holds the entire NELF complex together (Fig. 1D)^21–23^.

We hypothesized that the evolution of NELF proteins was associated with the gain of pausing in eukaryotes. To test this hypothesis, we used BLASTp to identify potential orthologs of the human NELF subunits among a group of 30 organisms representative of key eukaryotic taxa (Fig. 1E; **fig. S2**; **fig. S3**). We found that NELF-B and -C/-D are widely distributed in eukaryotes, suggesting that the core NELF subunits were present in a shared common ancestor of all eukaryotes. Both subunits were secondarily lost in yeast, *S. pombe and S. cerevisiae*, as well as in the nematode *C. elegans* and in land plants. NELF-A is present in some unicellular eukaryotes; a strong match to the metazoan protein first appeared in a common ancestor of Ichthyosporea and metazoans. We only found strong evidence for NELF-E in metazoans, and the most parsimonious model is that NELF-E evolved early in Metazoa or just before the transition to multicellularity. Collectively, these findings demonstrate a strong association between the evolution of NELF proteins and paused polymerase near TSSs.

To test whether other factors besides NELF were associated with the evolution of paused Pol II, we examined the evolutionary conservation of other proteins linked to pausing. Most other proteins implicated in the early steps of transcription elongation, including PAF1, DSIF (SPT4, and SPT5), the positive transcription elongation factor (P-TEFb; CDK9, cyclins [human Cyclin-T1, Cyclin-T2]), and 7SK (MEPCE, LARP7) are deeply conserved among eukaryotes (**Fig. S2; Fig. S3**), with some structural conservation extending back to archaea^24^. Thus, proteins responsible for the release from pause in metazoans (especially P-TEFb and PAF1) were part of the ancestral eukaryotic transcription complex, and evolved before the high pausing indices found in metazoans. The sole exceptions were the HEXIM proteins (HEXIM1 and HEXIM2), which are part of the 7SK complex that works in preventing P-TEFb-mediated pause release^25^. The most parsimonious model is that HEXIM proteins evolved in a common ancestor of Metazoa, perhaps coincident with NELF-E, as an additional checkpoint to increase the residence time of paused Pol II or to regulate pausing in metazoans.

Our finding that NELF and HEXIM protein evolution were uniquely associated with polymerase pausing led us to suspect that gains of specific NELF and HEXIM protein subunits may have resulted in incremental alteration of the residence time of paused Pol II along the animal stem lineage. To determine how the addition of multiple NELF subunits affected the strength of pausing, we compared pausing indexes between species with a different complement of NELF or HEXIM subunits. Species containing NELF-B and -C/-D (which we refer to as the “core” NELF complex) have higher pausing indexes than species without any NELF subunits (Fig. 1F; **Fig S4**). The addition of NELF-A increased pausing indexes to the same order of magnitude observed in metazoan model organisms (files and mammals). Thus, NELF-B and -C/-D are sufficient for pausing, but the addition of NELF-A, NELF-E, and HEXIM proteins correlates with higher pausing indexes and suggests the derived proteins may act together with the core NELF complex to fine-tune the function of paused RNA polymerase (**Fig. S4D**).

### Organisms without NELF show different types of pausing behavior

**Figure 2:**
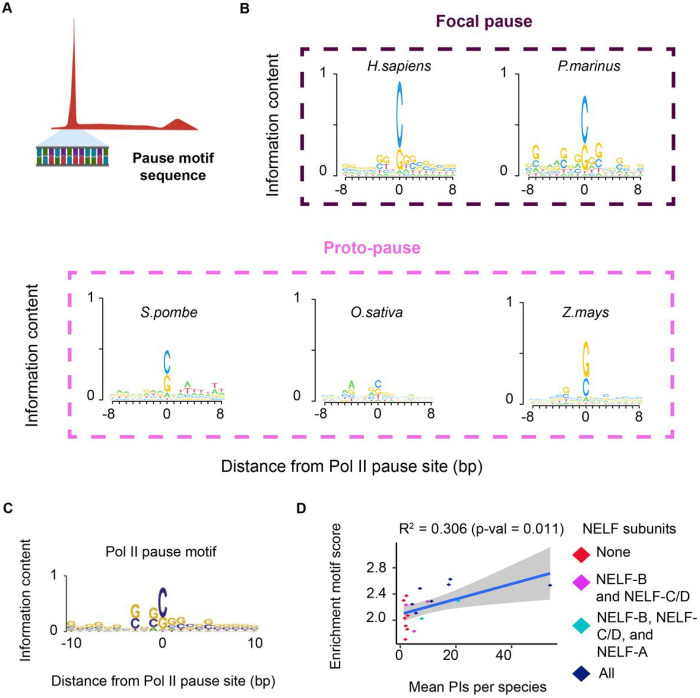
Genomic features are associated with pausing. (A) Schematic of motif search at the Pol II pause site. (B) DNA sequence motif under the active site of paused Pol II in the indication organisms with a focal pause or a proto-pause. The size of each base is scaled by information content. (C) Pause motif sequence as published in Watts *et al*., Am J Hum Genet., 2019. (D) Scatter plot denotes the enrichment of the motif score relative to flanking DNA and the mean pausing index in each species. Each dot is colored by the number of NELF subunits found in each sample. We fit a linear regression to derive the R^2^ and the p-value.

In some unicellular organisms, which do not have all four of the NELF subunits found in metazoans, we observed that Pol II moved slowly through the first 30–100 bp after the TSS. We hypothesized that this “proto-pause” may serve as an ancestral substrate pre-dating the highly focused, long-duration Pol II pausing observed in extant metazoans. In some cases, we observed examples of extreme phenotypes in which Pol II moved slowly despite having a complete absence of the NELF core complex. For instance, *Z. mays*, *S. pombe*, and *O. sativa* all display an accumulation of Pol II near the TSS, despite having lost both NELF-B and NELF-C/D. This extreme example of a proto-pause appears in a similar location as a canonical pause (or just downstream), but does not have the same magnitude of pausing index (Fig. 1C). To explore why some extant species have a proto-pause, despite not containing any of the NELF subunits, we examined the DNA sequence under the quartile of genes with the strongest positioned proto-pause in each organism (Fig. 2A). Consistent with previous work^11,26–30^, metazoan organisms show a well-defined pause motif, which is also present in three organisms that show a proto-pause, including *Z. mays, S. pombe,* and *O. sativa* (Fig. 2B). These observations may suggest that a pause DNA sequence motif contributes to a transient slowdown of Pol II at this position in organisms that have lost the core NELF subunits, NELF-B and NELF-C/D.

To determine whether the pause motif was associated with pausing index variation across all 20 species, we examined the enrichment of the human pause motif near the pause position (Fig. 2C). Despite the pause motif we used being derived from humans ^28^, we nevertheless found that it explained variation in the pausing index across all organisms surprisingly well (R^2^ = 0.306, *p* = 0.011; Fig. 2D; **fig. S5A-F**). Conversely, the DNA sequence motif of the TATA box and Initiator were not correlated with pausing index (**fig. S5G-H**). Altogether, our data support the idea that a pause sequence motif, featuring a C (or possibly G) in the Pol II active site at the pause position, serves as an ancestral step limiting the rate of transcription after initiation and can be linked to the formation of a proto-pause. This pause-associated DNA sequence alongside other chromatin factors, such as the position of the +1 nucleosome and the rate at which the P-TEFb subunit CDK9 phosphorylates the early elongating Pol II complex, may then be sufficient to explain much of proto-pause formation in species such as *S. pombe*^9^.

### Loss of core NELF-B impacts chromatin localization of NELF-E and alters Pol II pausing

**Figure 3:**
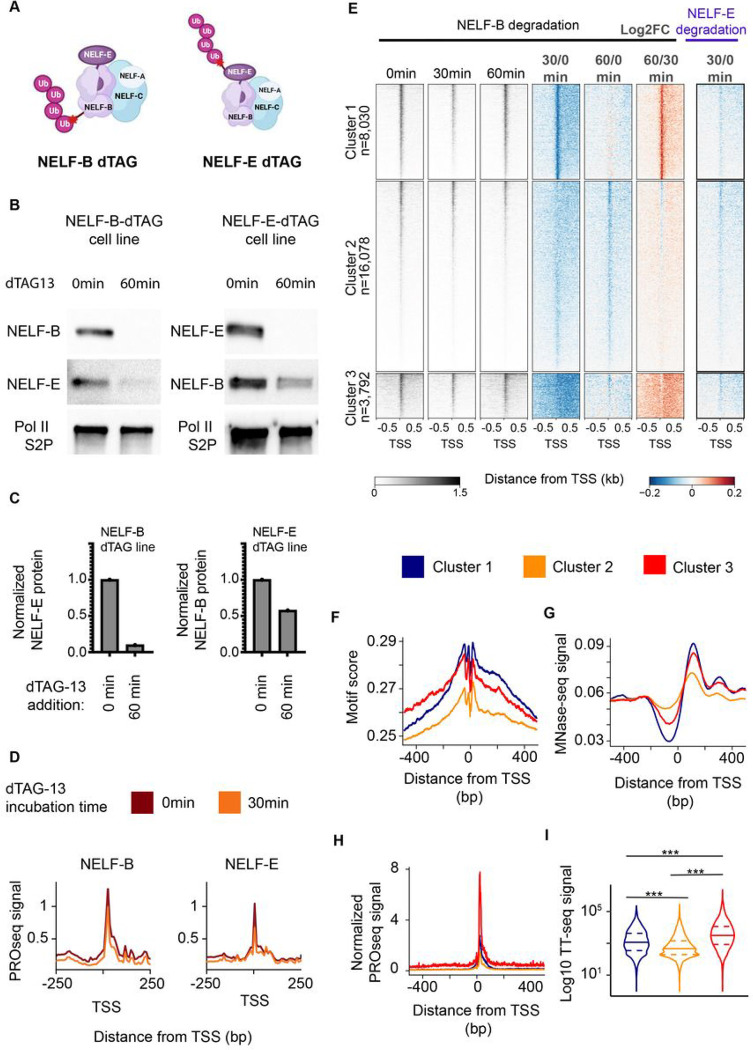
NELF degradation destabilized RNA Pol II pausing. (A) Schematic of NELF-B or NELF-E degradation mESC cell lines. (B) Western blots depict NELF-B, NELF-E and Pol II after the degradation of either NELF-B (left) or NELF-E (right) using 500nM dTAG-13. (C) Quantification of NELF-E western blot signal after NELF-B degradation (left) and NELF-B after NELF-E degradation (right). (D) Meta profiles of PRO-seq signal at 0min (red) and 30min (orange) after the degradation of NELF-B (left) or NELF-E (right). (E) Heat maps of spike-in normalized PRO-seq signal after NELF-B or NELF-E degradation (left). Log2 fold changes of normalized PRO-seq signal relative to untreated controls are also depicted. (F-H) Pause motif enrichment scores (F), Meta profile of MNase-seq signal (G) and normalized PRO-seq signal (H) are depicted for three clusters of genes defined in panel (E). (I) Violin plots of log10 TT-seq signal in the three clusters defined in (E). A two-sided Mann-Whitney test was used to compute p-values. (***) defines p-values < 2.2e-16

Our analysis of NELF evolution shows that the core NELF subunits, NELF-B and -C/D, evolved earlier than the ancillary subunits, NELF-A and -E. To test the functional impact of core and ancillary subunits in mammalian cells, we generated FKBP12-homozygously tagged mouse embryonic stem cell (mESC) lines that rapidly degrade either NELF-B (13) or NELF-E after treatment with the small molecule dTAG-13 (Fig. 3A–B^7,31^). The NELF-B dTAG was reported and validated in a recent paper^7^, while the NELF-E dTAG cell line is novel here. We verified that the FKBP12-tagged NELF-E protein was properly localized and that NELF-E was nearly undetectable within 30 min after the addition of 500 nM dTAG (**fig. S6; fig. S7**). We also verified that the rapid depletion of both NELF subunits decreased Pol II levels at the pause site following 30–60 min of dTAG-13 treatment, as measured by PRO-seq (Fig. 3E–F; **fig. S8A**). We hypothesized that loss of NELF-B would have a greater impact on NELF complex assembly on chromatin than loss of NELF-E due to the central role of NELF-B in the complex (Fig. 3A^21^). Consistent with our hypothesis, we observed that loss of NELF-B led to a decrease of the entire NELF-B/E sub-complex from chromatin, while a loss of NELF-E resulted in only a moderate reduction of 40% in NELF-B protein levels (Fig. 3B–C **left panel; fig. S7 fig. S6A-B**). These findings confirm that the functions of NELF-B and -E in mESCs mirror the structure and evolutionary history of these NELF subunits.

### Pol II recovery after prolonged NELF-B degradation mirrors a proto-paused-like state

Our PRO-seq data in the NELF-B cell line showed that many genes partially recovered Pol II at the pause site following 60 min of treatment (**fig. S8B-C**). To investigate the observed Pol II signal recovery, we first clustered genes based on their changes in Pol II loading between 30 and 60 min of dTAG treatment (Fig. 3E, clusters 1, 2, and 3). Cluster 1 showed a localized recovery of PRO-seq signal near the position of the canonical pause. Cluster 2 showed no indication of recovery and, relative to the other clusters, it was enriched in transcribed enhancer sequences (**fig. S9A**). And, cluster 3 exhibited a recovery of Pol II further into the gene body in a similar position as the slowdown of Pol II observed in *S. pombe* and *O. sativa*, potentially near the location of the +1 nucleosome, as reported by a previous study^32^.

We hypothesized that after the depletion of NELF, DNA sequences associated with the proto-pause in organisms without NELF-B may be sufficient to re-establish some paused Pol II. We looked for enrichment of the DNA proto-pause motif at loci that exhibited recovery of the paused state after NELF depletion. We found both higher enrichment of the pause motif and better positioning of the +1 nucleosome in clusters 1 and 3 when compared to cluster 2 (Fig. 3F–G; **fig. 9B**). Interestingly, the main difference between clusters 1 and 3 was that genes in cluster 3 had higher initiation rates, as determined by both TT-seq^33^ and a computational modeling approach analyzing steady-state PRO-seq data^17^ (Fig. 3H–I; **fig. S9C**). Genes in cluster 3 also had much higher binding of some components of the pre-initiation complex and more clearly defined DNA sequence motifs that specify transcription initiation^34^ (**fig. S9D-E**), potentially consistent with higher initiation rates. Based on these results, we propose that clusters 1 and 3 partially recover Pol II near the pause due to a combination of DNA sequence and interactions with well-positioned nucleosomes. We also speculate that genes in cluster 3 recover in a more downstream position as a result of a higher rate of initiation. Greater initiation rates at these genes may lead to an accumulation of Pol II at the start of the gene that causes polymerases to be pushed downstream due to interactions between newly incoming Pol II. In sum, we found that after NELF depletion, Pol II signal resembles the pattern found in proto-paused organisms that have lost the core NELF subunits. Furthermore, this proto-paused-like state is associated with the same DNA sequence features and the presence of strongly positioned nucleosomes.

### Pol II pausing allows transcription factors to regulate pause release

We speculate that the evolution of a focal pause was required for the evolution of a system that could control gene expression by releasing paused Pol II. In metazoans, sequence-specific transcription factors can modulate pause release and thereby tune the level of gene expression^5,6,35–38^. The factors that are responsible for pause release (e.g., p-TEFb) are conserved in all eukaryotes (**fig. S2; S3**), pointing to the critical role of release (or an analogous step in early elongation) in eukaryotic organisms^10^. After the depletion of NELF-B, we observed paused Pol II “creeping” across the first couple of kilobases of the gene body (**fig. S10**), similar to observations made in *S. pombe*, which has no NELF^7,10^. As a result, Pol II which needs to be released from pause by p-TEFb is no longer in a fixed location, in proximity to promoter-bound transcription factors.

**Figure 4:**
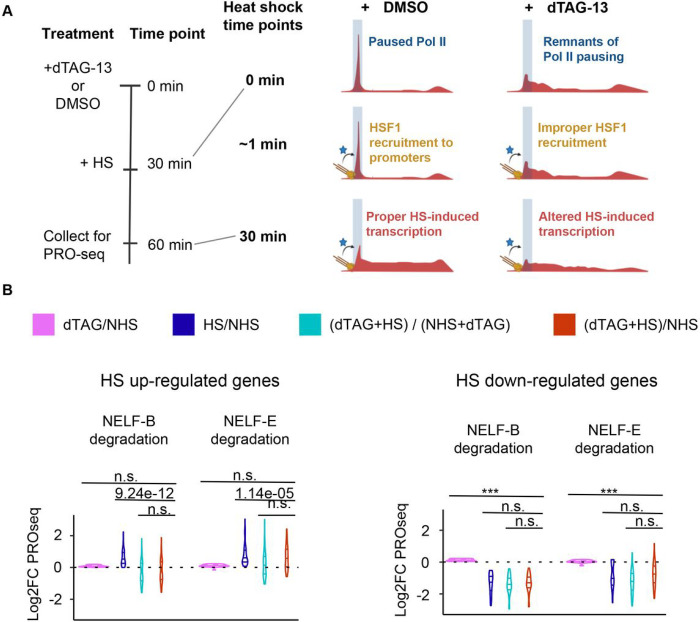
Removing paused Pol II prevents activation of genes by HSF1 after HS stimulation. (A) Time course of dTAG-13 drug treatment followed by heat shock (HS) (left) and cartoon depicting mechanisms of HSF1 action on Pol II pausing (right). HSF1 is depicted in yellow, while other co-factors that assist in pause release are depicted in blue. (B) Violin plots of log2 fold changes in PRO-seq signal for HS-upregulated (top) and downregulated (bottom) genes. A two-sided Mann-Whitney test was used to compute p-values, where n.s. Defines non-significant p-values, and (***) p-values < 2.2e-16.

We hypothesized that the downstream redistribution of Pol II after NELF-B depletion would prevent the targeted regulation of gene expression by transcription factors acting to release paused Pol II into productive elongation. To test this hypothesis, we turned to the well-studied heat shock system, where the transcription factor heat shock factor 1 (HSF1) activates transcription of a core group of a few hundred genes following heat stress by the release of paused Pol II^36,39,40^. We asked whether HSF1 could release paused genes as efficiently following the depletion of NELF-B and -E in mESCs (Fig. 4A). We first identified genes that were up- and down-regulated using a regular heat shock experiment in mESCs. Our analysis confirmed the induction of a core group of heat shock-responsive genes^36,39,40^, despite some differences in basal gene expression between NELF-B and NELF-E cell lines (**fig. S11; fig. S12A-D**). Although many classical up-regulated genes were properly up-regulated following the depletion of NELF-B and NELF-E, heat shock (HS)-dependent genes on average had a lower induced fold-change following NELF-B depletion (**fig. S12; fig. S13E**). Thus, Pol II redistribution after NELF-B depletion does prevent HSF1 from acting efficiently as a transcriptional activator.

To rule out the possibility that our observed differences in HSF1-dependent gene activation were driven by changes in gene expression following dTAG-13^7^, including the accumulation of Pol II trickling into the gene body (**fig. S10**), we focused our analysis on the gene body downstream from NELF-induced Pol II trickling regions. We also excluded genes with altered gene body density following dTAG-13 treatment in either cell line (**fig. S13F; see** Methods). For the remaining genes, we noted a clear defect in the HS-induction of up-regulated genes, but not in HS-repression at down-regulated genes, consistent with a model in which HSF1 failed to adequately release Pol II after NELF depletion (Fig. 4B). The up-regulation defect was more prominent following the depletion of NELF-B than NELF-E (unpaired Mann-Whitney, p-value = 2.8e-4) (Fig. 4B), potentially consistent with a more direct role for NELF-B in the formation of a focal pause. Interestingly, many of the most highly HS-induced genes did not show a large defect in up-regulation as seen here (e.g. *Hspa1b*, *Hsp1h1*; **fig. S12**) and in a previous study^32^. The high rate of firing at these genes may be associated with a high concentration of p-TEFb, resulting in a higher probability of releasing Pol II in the right location before it trickles away from the promoter. In contrast, the more moderately induced and highly paused HS genes are firing less frequently and the trickling of paused Pol II to more downstream locations may prevent their proper activation (**fig. S13**). Altogether, these findings support our model in which the evolution of pausing facilitated the ability for transcription factors to act on pause-release, providing an additional step to more tightly control gene expression.

## Discussion

Our work offers mechanistic insights into how new regulatory complexity evolved by enabling targeted regulation of a preexisting step in the transcription cycle through the evolution of a focal promoter-proximal pause. We propose that the recruitment of P-TEFb and PAF1, which cause pause-release in metazoans, actually serve a more general role that is necessary at all genes in all eukaryotic organisms, regardless of whether the organism has a long-lived focal pause. The evolution of a “focal” pause collapsed the substrate for this step in transcription to a single location at each gene and increased the pause residence time. The degree to which Pol II slows down at the pause position, which progressively increased in the branches leading to metazoans, appears to be affected by the evolution of NELF-E, NELF-A, and HEXIM proteins. Together these evolutionary innovations collapsed a rate-limiting step in all eukaryotes into a single position in metazoans. A centralized location for paused Pol II allows transcription factors to catalyze the release of Pol II into productive elongation, by providing a focused and promoter-proximal target adjacent to transcription factor binding sites, as shown here in the case of HSF1. This innovation provided a new rate-limiting step in transcription that could be targeted for gene-specific regulation. The evolution of additional regulatory complexity may have helped to enable the evolution of complex, multicellular metazoan organisms.

## Methods

### Data Availability

Tables in CSV format can be downloaded from: https://github.com/alexachivu/PauseEvolution_prj

Data generated in this study can be found in Gene Expression Omnibus at: GSE223913.

### Code Availability

Custom code for analyzing sequencing data can be found on GitHub under: https://github.com/alexachivu/PauseEvolution_prj/

### Experimental methods

#### Sample collection:

##### E. coli:

An overnight culture of E. coli MG1655 was subcultured in 50 mL LB and grown at 37°C to OD 600 = 0.95. 5 mL aliquots were pelleted by centrifugation at 3000 × g. Pellets were permeabilized, washed, and flash-frozen as described in ^44^.

##### H. mediterranei:

ATCC 33500 was grown for 48 hours at 35 °C in ATCC Medium 1176. 12.5 mL culture was centrifuged, and the cell pellet was resuspended in 3 mL cold non-yeast permeabilization buffer. To increase permeabilization of archaeal cells, the cell suspension was split into 3 × 1 mL aliquots in screw-cap tubes and combined with 400 μL sterile 0.5 mm glass beads. Cells were subject to bead-beating for 3 cycles of 2 minutes vortexing, 2 minutes on ice. Supernatants were transferred to 1.5 mL tubes, centrifuged to collect cell contents, and washed twice by resuspension in 500 μL storage buffer. Cells were resuspended in a final volume of 50 μL storage buffer and snap-frozen. The permeabilization and storage buffers were the same as reported previously ^44^, and include: ATCC Medium 1176 recipe (1 L), 156 g NaCl, 13 g MgCl 2 × 6H 2 O, 20 g MgSO 4 × 7H 2 O, 1 g CaCl 2 × 2H 2 O, 4 g KCl, 0.2 g NaHCO 3, 0.5 g NaBr, 5 g yeast extract, 1 g glucose. After mixing components, the pH was adjusted to 7.0 and the buffer was autoclaved.

*Sea Lampreys (Petromyzon marinus)* were obtained from Lake Michigan via the Great Lakes Fisheries Commission and maintained under University of Kentucky IACUC protocol number 2011–0848 (University of Kentucky Institutional Animal Care and Use Committee). For tissue sampling, animals were euthanized by immersion in buffered tricaine solution (1.0 g/l), dissected, and tissues were immediately frozen in liquid nitrogen. We analyzed muscle samples taken from the flank of one male and one female.

##### Sea urchin (S.purpuratus):

All of the animal rearing and downstream processing use the same protocols as ^45^. Biological replicates of 20 hour blastula embryos were raised at 15°C in 0.2 um filtered sea water. The embryos were then spun down at 500 G and 0°C for 3 minutes and the pellets were flash-frozen in liquid nitrogen, stored at −80°C and shipped in dry ice.

##### D. iulia:

Wing tissues were sampled from Day 3 pupae derived from Costa Rican stock following standard protocols (e.g. ^46^ and ^16^). Wing tissues were dissected from pupae in cold PBS, after which nuclei were extracted in cold PBS using a dounce homogenizer. Nuclei were spun down and resuspended in nuclei storage buffer before flash freezing.

*Capsaspora owczarzaki* (strain ATCC 30864) was cultured axenically at 23°C in ATCC medium 1034 (modified PYNFH medium) in tissue culture-treated flat-bottomed polyethylene tissue culture flasks. Confluent cells were harvested by centrifugation (5000 × g, 5 minutes), and the pellet flash-frozen and stored at −80°C. For the isolation of intact nuclei, cells were harvested as before; the pellet was washed twice with phosphate-buffered saline (PBS), resuspended in 1ml of 2x Lysis Buffer (for 2x buffer: 10mM Tris-Cl pH 8.0; 300mM sucrose; 10mM NaCl; 2mM MgAc2;6mM CaCl2; 0.2% NP-40) and incubated on ice for 18 minutes. The resulting lysate was centrifuged (5000 × g, 5 minutes), and the pellet containing nuclei was washed once with 1ml Wash Buffer (10mM Tris-Cl pH 8.0; 300mM sucrose; 10mM NaCl; 2mM MgAc2). The nuclei were pelleted once more, resuspended in 1ml Storage Buffer (50mM Tris-Cl pH 8.0; 40% glycerol; 5mM CaCl2; 2 mM MgAc2), and stored at −80°C. For each buffer, 2 PhosStop^™^ phosphatase inhibitor tablets (Roche), 1mM PMSF, 50 μg Pepstatin A, 56 mg sodium butyrate, and 1 cOmplete^™^ Protease Inhibitor Cocktail tablet (Roche) per 50ml buffer were added immediately prior to use.

*Creolimax fragrantissima* and *Sphaeroforma arctica* were cultured axenically in BD Difco^™^ Marine Broth 2216, in tissue culture-treated flat-bottomed polyethylene tissue culture flasks at 12°C; confluent cells were harvested by centrifugation, and the pellet flash-frozen and stored at −80°C.

*Dictyostelium discoideum* AX3 wildtype cells were cultured axenically in HL-5 (Formedium) on untreated polystyrene petri dishes at 22°C. Confluent cells were resuspended in fresh media and centrifuged at 300×*g* for 5 min. The pellet was flash frozen and stored at −80°C.

##### Nematostella vectensis:

Adult Nematostella were reared in 1/3 strength artificial seawater at 18°C in dark conditions. Spawning was induced using the protocol described in ^47^. Adult males and females were induced to spawn in small glass bowls, and fertilized egg masses were removed and cultured in small glass bowls at 25°C. Swimming gastrula/early polyp stage animals were harvested for nuclei isolation.

##### Mouse embryonic stem cell (mESC) cell culture:

E14 mESCs (ATTC) were cultured on 0.1% gelatin-coated (Millipore) tissue culture-grade plates in a humidified 37°C incubator with 5% CO2. The culture medium consisted of DMEM (Gibco) supplemented with 2 mM L-glutamine (Gibco), 1× MEM nonessential amino acids (Gibco), 1 mM sodium pyruvate (Gibco), 100 U/mL penicillin/100 U/mL streptomycin (Gibco), 0.1 mM 2-mercaptoethanol (Gibco), 15% fetal bovine serum (Gibco), and 1000 U/mL recombinant leukemia inhibitory factor (LIF). Genetic editing and experiments were performed using cells at passages 10–20.

##### Generation of NELFB and NELFE mESCs:

both cell lines were generated using an identical approach to endogenously and homozygously tag the C-terminus of each protein with FKBPF36V tag. The NELFB line has been previously described, and the NELFE line was generated for this study. The methods below describe the NELFE line generation, for more details of NELB line, please refer to ^7^.

##### Plasmid Generation:

To target the *nelf-e* genes, two plasmid constructs were generated:

1. Cas9 vector to target the C terminus of Nelfe gene: PX459 vector (Addgene 62988) was digested using BbsI-HF (NEB) and single guide RNA targeting Nelfe was annealed (Ran et al. 2013)..
2. Homology-directed repair (HDR) vector containing the insert FKBPF36V tag, 2× HA tag, self-cleaving P2A sequence, and puromycin resistance, flanked by 1-kb Nelfe HDR sequences: The insert was obtained from pCRIS-PITCHv2-dTAG-Puro (Addgene 91796) (Nabet et al. 2018). The plasmid backbone (pBluescript), Nelfe HDR sequences, and the insert were amplified using Q5 polymerase (NEB), and the plasmid was constructed using NEBuilder HiFi DNA assembly (NEB). All oligos used are available in the table below.

**Table:** 

Name	Sequence	function
Nelfe_sgRNA	CACCGTGTGTACAGTGACGATCTAT	sgRNA following the ‘CACC’ for PX459 insertion
Nelfe_sgRNA’	AAACATAGATCGTCACTGTACACAC	Complement of sgRNA oligo for PX459 insertion
HDR-template plasmid pieces		
Nelfe_LA_F	acggtatcgataaagccatttggaaaaacag	Apmplify left homology arm
Nelfe_LA_R	cacctgcactccatcgtcactgtacacaatc	Apmplify left homology arm
Nelfe_RA_F_Puro	cccggtgcctgactataggaaaccttgtggatg	Apmplify right homology arm
Nelfe_RA_R	aactagtggatccaggtcaaagatgcctctg	Apmplify right homology arm
Nelfe_dTAGpuro_F	gtacagtgacgatggagtgcaggtggaaaccatctc	Amplify FKBP (F36V) tag
Nelfe_dTAGpuro_R	aggtttcctatagtcaggcaccgggcttgcg	Amplify FKBP (F36V) tag
Nelfe_BB_F	catctttgacctggatccactagttctagagc	Amplify pBluescript backbone
Nelfe_BB_R	ttccaaatggctttatcgataccgtcgacctc	Amplify pBluescript backbone

##### Generation of Nelfe-dTAG mESCs:

3 million cells were transfected with 10 μg of PX459-Nelfe_sgRNA and 10 μg of Nelfe_left-FKBPF36V-2xHA-P2A-PURO-Nelfe_right using the Lonza P3 primary cell 4D-Nucleofector X 100-μL cuvettes. The transfected cells were plated on a 10-cm dish coated with mouse embryonic fibroblasts (MEFs). 48 hours after transfection, correctly targeted cells were selected for in 6 μg/mL Blasticidin for 5 days. Surviving cells were split into 1000 cells per 10-cm dish and maintained for 9 days under puromycin selection. Surviving clones were picked and expanded under a stereomicroscope and genotyped for the insert.

##### dTAG drug treatment:

The dTAG-13 reagent (Bio-Techne: https://www.bio-techne.com/p/small-molecules-peptides/dtag-13_6605) was reconstituted in DMSO (Sigma) to a final concentration of 5 mM. The dTAG-13 solution was diluted in culture medium to 500 nM and added to cells for the indicated time period during medium changes.

##### Immunofluorescence:

Cells plated on u-Slide eight-well plates (Ibidi) were washed with PBS+/+ and fixed in 4% PFA (Electron Microscopy Sciences) in PBS+/+ for 10 min at room temperature. Cells were subsequently washed twice with PBS+/+, followed by wash buffer and 0.1% Triton X-100 (Sigma) in PBS+/+, and permeabilized in 0.5% Triton X-100 (Sigma) in PBS+/+ for 10 min. Then blocked with 3% donkey serum (Sigma) and 1% BSA (Sigma) for 1 h at room temperature. Cells were incubated with primary antibodies in blocking buffer overnight at 4°C (antibodies and concentrations are listed in Supplemental Table S1). Then, they were washed three times in wash buffer and incubated with suitable donkey Alexa Fluor (1:500; Invitrogen) for 1 h at room temperature. Finally, cells were washed three times with wash buffer, with the final wash containing 5 μg/mL Hoechst 33342 (Invitrogen), and imaged.

##### Imaging:

Fixed immunostained samples were imaged using a Zeiss LSM880 laser scanning confocal microscope. An air plan-apochromat 20×/NA 0.75 objective was used. Images represent a 2D plane correlating to the monolayer of cells in culture. No further image processing was performed.

##### Western blotting:

Cells were harvested and lysed by adding 350 μL of lysis buffer containing 1× cell lysis buffer (Cell Signaling), 1 mM PMSF (Cell Signaling), and cOmplete Ultra protease inhibitor (Sigma) to a 90% confluent six-well dish (Falcone) after washing with PBS−/−.

The harvested cells were incubated on ice for 5 min, scraped and collected then sonicated for 15 sec to complete lysis and then spun down at 12,000g for 10 min at 4°C. The supernatant was collected, and protein concentration was measured using Pierce BCA protein assay kit (Thermo). Samples were prepared by mixing 10 to 20 μg of protein with Blue loading buffer (Cell Signaling) and 40 mM DTT (Cell Signaling), followed by boiling for 5 min at 95°C for denaturation. Cellular compartment fractions were prepared using subcellular protein fractionation kit (Thermo) following the manufacturer’s instructions.

The samples were run on a Bio-Rad Protean system and transferred to a nitrocellulose membrane (Cell Signaling) using transblot semidry transfer cells (Bio-Rad) following the manufacturer’s instructions and reagents. The nitrocellulose membrane was briefly washed with ddH2O, stained with Ponceau S (Sigma) for 1 min, and washed three times with TBST (0.1% Tween 20 [Fisher] in TBS) to check for transfer quality and serve as a loading control. Then it got blocked with 4% BSA in TBST for 1 h at room temperature and incubated with primary antibodies diluted in blocking buffer overnight at 4°C. The membrane was then washed three times with TBST, incubated with secondary antibodies in blocking buffer for 1 h, and washed three times with TBST. Last, the nitrocellulose membrane was incubated with ECL reagent SignalFire for 1–2 min and imaged using a ChemiDoc (Bio-Rad).

The following antibodies were used in this paper:

**Table:** 

Antibody	Source	Identifier	Application	Conc.
anti-Histone H3	Cell Signaling	Cat# 4499, RRID: AB_10544537	Western	1:2000
anti-RNA pol II S2P	Abcam	Cat# ab193468, RRID: AB_2905557	IF	1:500
anti-NELFE	Abcam	Cat# ab170104, RRID: AB_2827280	Western	1:1000
anti-COBRA1/NELFB	Cell Signaling	Cat# 14894, RRID: AB_2798637	Western	1:1000
anti-HA	Abcam	Cat# ab130275, RRID: AB_11156884	IF	1:500
anti-HA	Cell Signaling	Cat# 3724, RRID: AB_1549585	Western	1:1000
anti-b-actin	Cell Signaling	Cat# 3700, RRID: AB_2242334	Western	1:2000
anti-DSIF/Spt5	BD Biosciences	Cat# 611106, RRID: AB_398420	Western	1:2000

##### Heat shock experiments on mESCs:

Heat shock was administered as described in recent work from the Lis lab ^36,40^. We started the heat stress after 30 min of dTAG-13 treatment, which corresponds to the maximal depletion of paused Pol II based on PRO-seq data.

We performed the analysis of the HS data in two different ways:

- On a first analysis, by calling gene expression changes using DEseq (log2foldChange > or < 0, and padj < 0.05) between a regular HS and NHS experiment. Then, we plotted log2 fold changes of (HS+dTAG)/(NH) and (HS+dTAG)/(NHS+dTAG) at these pre-defined HS up-regulated or down-regulated genes coordinates.
- For a second approach, we considered the effect that NELF-B depletion has on Pol II trickling into gene bodies. To eliminate any biases from increased PRO-seq signal downstream from the TSS due to the dTAG-13 treatment alone, we re-analyzed our data after removing the first 3 kb downstream from the TSS and we focused on genes that remain unchanged following dTAG-13 treatment, but are either up or down-regulated after HS (**fig. S13F**). We confirmed a slight up-regulation of genes in the vicinity of the TSS after dTAG-13 treatment by plotting the correlation between the log2 fold change of dTAG-13 treatment after NELF-B degradation (**fig. S10**). We used deeptools to compute log2 fold change bigwigs and plot heat maps.

Both of these analyses confirmed a defect in up-regulation across many HS-dependent genes. In the second analysis, we observed that a significant number of genes that were meant to show HS-dependent upregulation failed to reach their full transcription potential in the absence of NELF-B (Fig. 4C **left; fig. S13A**). The same effect was also observed, though in far fewer genes in the absence of NELF-E (Fig. 4C **right; fig. S13B**). We noted no defect in down-regulated genes using this analysis approach.

##### PRO-seq library prep:

PRO-seq or ChRO-seq ^11,48^ libraries were prepared from snap-frozen cell pellets following the protocol described in ^44^. All PRO-seq libraries were evaluated for data quality and sequencing depth using PEPPRO ^49^. Data and data quality are shown in (**fig. S14; Table 1**).

### Computational analyses

*In this paper we refer to PRO-seq, GRO-seq, and ChRO-seq as **PRO-seq**.

#### Mapping and processing PRO-seq data:

Single and paired-end reads of PRO-seq data were aligned to its reference genome using the proseq2.0 pipeline from the Danko lab (https://github.com/Danko-Lab/proseq2.0) using the following parameters: -RNA5=R1_5prime --RNA3=R2_5prime --ADAPT1=GATCGTCGGACTGTAGAACTCTGAACG --ADAPT2=AGATCGGAAGAGCACACGTCTGAACTC --UMI1=4 --UMI2=4 --ADD_B1=6 --ADD_B2=0 --thread=8 --map5=FALSE. Library processing included adapter trimming using cutadapt, PCR deduplication (where UMIs are present) using printseq-lite.pl, followed by mapping to the reference genome using BWA. Mapped BAM files were then trimmed either to the 3’-end of the RNA (to map the location of RNA Pol II) or the 5’-end (to map the beginning of the RNA) and the 1bp position was converted to bedGraphs and BigWigs. PRO-seq libraries were also RPM normalized to account for differences in sequencing depth.

Data was mapped to the following reference genomes:

- E.coli:escherichia_coli_mg1655_01312020 (https://www.ncbi.nlm.nih.gov/nuccore/U00096.2)
- Haloferax: NZ_CP039139.1 (https://www.ncbi.nlm.nih.gov/nuccore/NZ_CP039139.1) and the plasmids included here: https://www.ncbi.nlm.nih.gov/genome/?term=txid523841
- D.discoideum: dicty_2.7 (https://www.ebi.ac.uk/ena/browser/view/GCA_000004695.1)
- A.thaliana: Arabidopsis_thaliana.TAIR10.dna.toplevel
- Z.mays:GCF_902167145.1_Zm-B73-REFERENCE-NAM-5.0 (https://www.ncbi.nlm.nih.gov/assembly/GCF_902167145.1/)
- O.sativa:GCF_001433935.1_IRGSP-1.0_genomic.fna (https://www.ncbi.nlm.nih.gov/assembly/GCF_001433935.1/)
- C.owczarzaki: Capsaspora_owczarzaki_atcc_30864.C_owczarzaki_V2.dna.toplevel
- S.pombe: Schizosaccharomyces_pombe.ASM294v2.dna.toplevel
- S.cerevisiae: Saccharomyces_cerevisiae.R64-1-1.dna.toplevel
- S.arctica: Sphaeroforma_arctica_jp610.Spha_arctica_JP610_V1.dna.toplevel.fa.gz (https://www.ncbi.nlm.nih.gov/assembly/GCF_001186125.1/)
- C.fragmatissima: Creolimax_fragrantissima.genome (ncbi.nlm.nih.gov/assembly/GCA_002024145.1/)
- N.vectensis: nemVec1
- C.elegans: ce6 (https://hgdownload.soe.ucsc.edu/goldenPath/ce6/chromosomes/)
- D.pulex: dpulex_jgi060905 (http://wfleabase.org/prerelease/dpulex_jgi060905/genome-assembly/)
- D.iulia: published assembly in ^46^
- D.melanogaster:dm3 (http://genome.ucsc.edu/cgi-bin/hgTracks?db=dm3&chromInfoPage=)
- S.purpuratus: Spur_3.1 (https://www.ncbi.nlm.nih.gov/assembly/GCF_000002235.3/)
- P.marinus: petMar2 (https://www.ncbi.nlm.nih.gov/assembly/GCA_000148955.1/)
- M.musculus: mm10 (GRCm38)
- H.sapiens: hg19 (https://www.ncbi.nlm.nih.gov/assembly/GCF_000001405.13/)

#### Reannotation of transcription start sites:

We took PRO-seq mapped BAM files and ran it through the RunOnBamToBigWig tool developed in the Danko lab (https://github.com/Danko-Lab/RunOnBamToBigWig) to compute 5’prime mapped BigWigs (parameter for paired end data: --RNA5=R1_5prime; parameter for single end data: --SE_READ=RNA_5prime). Then, we used published gene annotations in each species, resized them to a 1kb window centered on the gene annotation start site, and computed the total number of 5’-prime mapped PRO-seq reads that fall within this interval using 10bp sliding windows. Last, to reannotate gene start sites, we took the start position of the 10bp window with the maximum number of 5’-prime PRO-seq reads. We used these annotated TSSs for all further analyses.

#### Computing Pausing indexes:

Pausing indexes were computed as the ratio between Pol II density in the pause region and gene body region. We defined the pause region as the interval between [ TSS-150, TSS+150 ]bp and the gene bodies as [ TSS+300, TES-300 ]bp (where TES = transcription end site). Genes shorter than 300bp were removed from the analysis.

#### Heatmaps and meta profiles:

We use DeepTools to functions (bigwigCompare, compute matrix, plotHeatmap, and plotProfile) to compute heatmaps and meta profiles of PRO-seq data. We also used DeepTools to cluster and compute correlations between the heat shock PRO-seq libraries as BAM files (bamCompare, and multiBamSummary, plotPCA, and plotCorrelation) ^50^.

Before running bigwigCompare, we generated combined BigWigs of the plus and minus PRO-seq data for each sample.


**bigwigCompare** --bigwig1 HS_plus.minus.bw --bigwig2 NHS_plus.minus.bw -o HS.NHS_log2FC_plus.minus.bw --outFileFormat bigwig --pseudocount 0.1 --operation log2 --skipNAs -p max/2 &



**computeMatrix** reference-point --referencePoint center -R regions.bed -S NHS_plus.minus.bw HS.NHS_log2FC_plus.minus.bw --samplesLabel “NHS” “HS / NHS”
--sortUsingSamples 1 \
-b 1000 -a 50000 \
--binSize 1000 \
--skipZeros -o Counts_GB.increase_log2FCs_NELF_B.gz -p max/2



**plotHeatmap** -m Counts_GB.increase_log2FCs_NELF_B.gz \
   -out Heatmap_Counts_GB.increase_log2FCs_NELF_B.pdf \
   --colorList “blue,white,red” \
   --heatmapHeight 10 \
   --heatmapWidth 3 \
   --zMin −2 --zMax 2 --missingDataColor 0 \
   --averageTypeSummaryPlot “mean”
**plotProfile** -m Counts_GB.increase_log2FCs_NELF_B.gz \
   -out Metaplot_Counts_GB.increase_log2FCs_NELF_B.pdf
**multiBamSummary** bins \
    --bamfiles ./*bam \
    --minMappingQuality 10 \
    -p max/2 \
    -out allTSS_QC_readCount_corr.npz \
    --outRawCounts allTSS_QC_readCount_corrRaw.tab
**plotCorrelation** \
    -in allTSS_Abood.PROseq_readCount_corr.npz \
    --corMethod spearman --skipZeros \
    --plotTitle “Spearman Correlation at annotated TSSs” \
    --whatToPlot heatmap --colorMap seismic --plotNumbers \
    -o allTSS_QC.Spearman.heatmap_readCounts.png \
    --outFileCorMatrix allTSS_QC.PROseq_readCount_Spearman.tab


#### Reciprocal BLASTp to compare transcription proteins across species:

To determine if human orthologs of key transcription machinery proteins (NELF complex; DSIF; PAF1; 7SK proteins) were present in other species, we performed a reciprocal BLAST using the rBLAST library (version 0.99.2). First, given a human protein, H, a BLASTp was performed against the protein database of a different organism (X). Sequences in species X that produced a BLASTp E-value lower than 1e-6, were considered candidates for a second BLASTp run. On the second BLASTp run, we performed a reciprocal BLAST search using the candidates in species X relative to the human proteome. If this reciprocal search yielded any valid hits passing the scoring same threshold of E-value < 1e-6, the protein was considered present in species X. This approach was repeated for all protein sequences in **fig. S2** and **S3**. For this analysis, we downloaded human protein sequences from UniProt, along with complete proteomes (.pep.all.fa files) of all species analyzed in this study from NCBI or Ensembl (only for *C. fragrantissima* and *D. iulia*).

Then, we used the following script to compare (https://github.com/alexachivu/PauseEvolution_prj/blob/main/Protein%20BLAST%20(BLASTp)) the human homologs of those proteins to the entire proteome of all species in this study. We provide the human protein sequences used in this reciprocal BLAST search here: https://github.com/alexachivu/PauseEvolution_prj/blob/main/Human.orthologs_sequences.fa

#### Defining DNA sequence motif under the pause:

The position of the pause site in each species was defined as follows. First, we utilized our reannotated TSS positions and created a 100bp window starting from the TSS: [ TSS, TSS+100 ]bp. Next, we designated the base with the maximum PRO-seq counts within this 100bp window as the pause site. Finally, we created either a 1kb or 20bp window centered around the identified pause site for further motif analyses.

#### Computing the enrichment of the metazoan pause motif across all species:

#1. We used bedtools getfasta to get DNA sequences at in a window centered on the pause site or on the TSS (the example below is provided for mouse data) bedtools getfasta -s -fi mm10.fa.gz -bed ./M.musculus_Pause.20bp.bed -fo M.musculus_Pause.20bp.out
#2. *We then run the MotifDicovery pipeline (*https://github.com/alexachivu/PauseEvolution_prj/blob/main/MotifDiscovery*) to discover DNA sequences associated with the pause site*
  R --vanilla --slave --args $(pwd) M.musculus_Pause.20bp.out
  M.musculus_Pause.20bp_SeqLogo.pdf < MotifDicovery.R
#3. For the motif enrichment analysis, we used a different script (https://github.com/alexachivu/PauseEvolution_prj/blob/main/MotifEnrichment) to compare the DNA sequences in each species with a human pause motif described in ^28^.

#### Differential analysis:

We performed differential analysis to quantify changes after heat shock, dTAG-13, and the dual treatment of heat shock and dTAG-13. To accomplish this, we run DEseq2. We used the total number of dm3 spike-in reads (divided by the mean of the spike-ins) as scaling factors.

#### Figure design:

We used BioRender to draw all of the illustrations and cartoons in this paper, with the exception of the schematic representing the relationships between species. The latter was prepared using Interactive Tree of Life (iTOL) v.6.7 ^51^ based on relationships depicted in ^52^ and edited in InkScape and Illustrator.

*An inventory of all functions used to process the PRO-seq data is deposited on GitHub at:* https://github.com/alexachivu/PauseEvolution_prj/tree/main

## Figures and Tables

**Figure 5:**
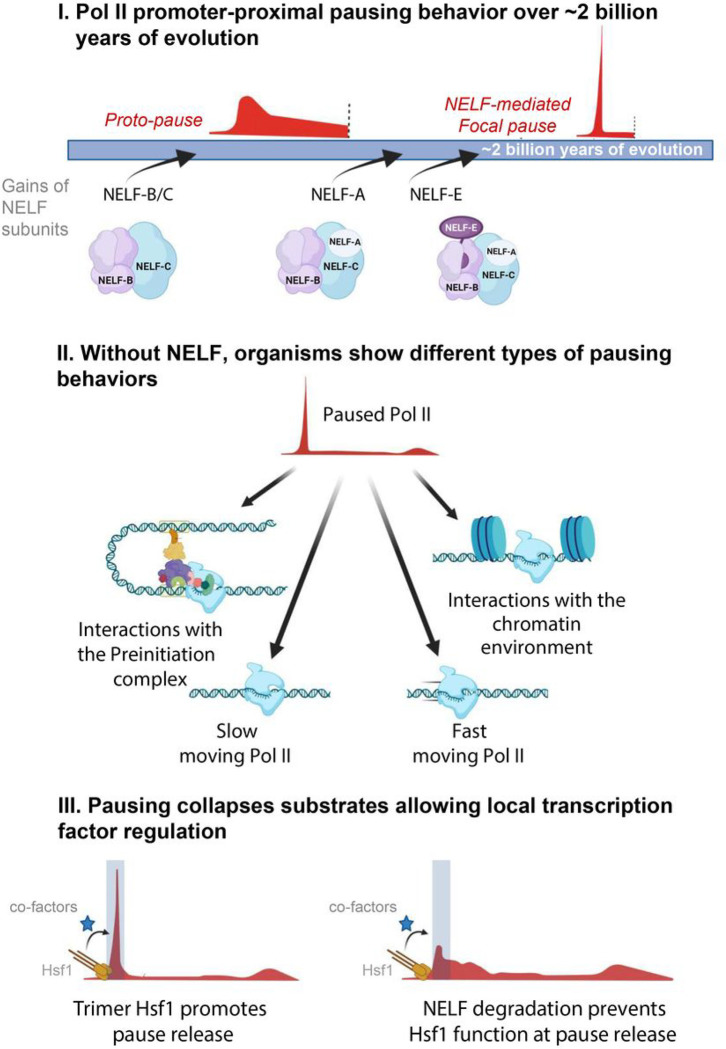
Summary model. The summary describes NELF protein complex evolution and assembly (I), pausing behaviors in the absence of NELF (II), and the influence of Pol II pausing on gene regulation by transcription factors (III). CA depotes Common ancestor.
